# Supplementary material for: Plasma Metabolomics Predicts Chemotherapy Response in Advanced Pancreatic Cancer
Source: Cancers (Basel). 2023 Jun 1;15(11):3020. doi: 10.3390/cancers15113020 (PMC10252041; doi:10.3390/cancers15113020)

Supplementary Figure S1

A

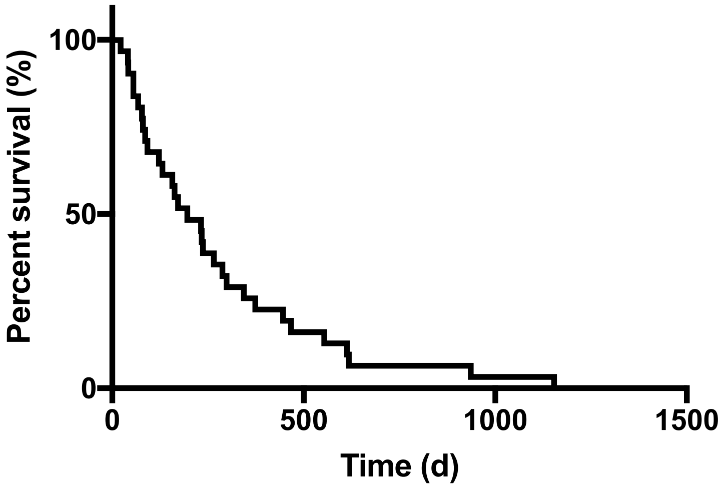

B

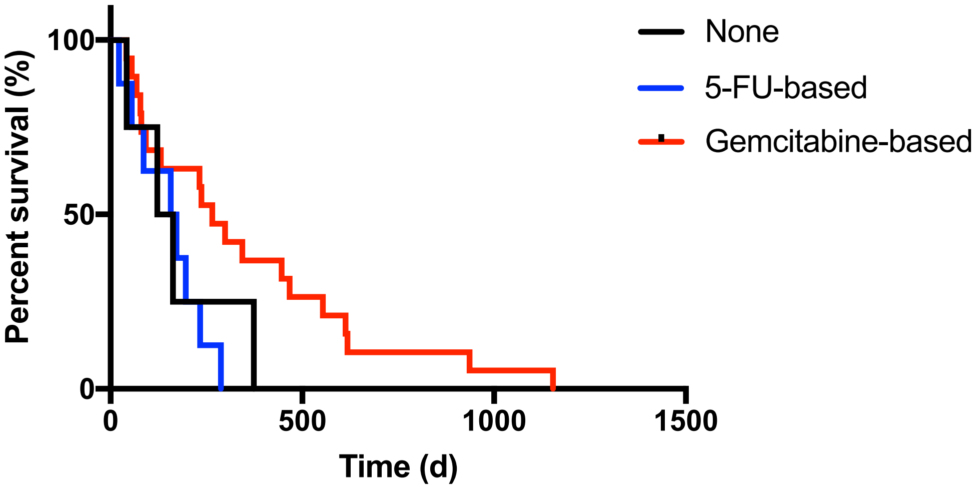

Supplementary Figure S2

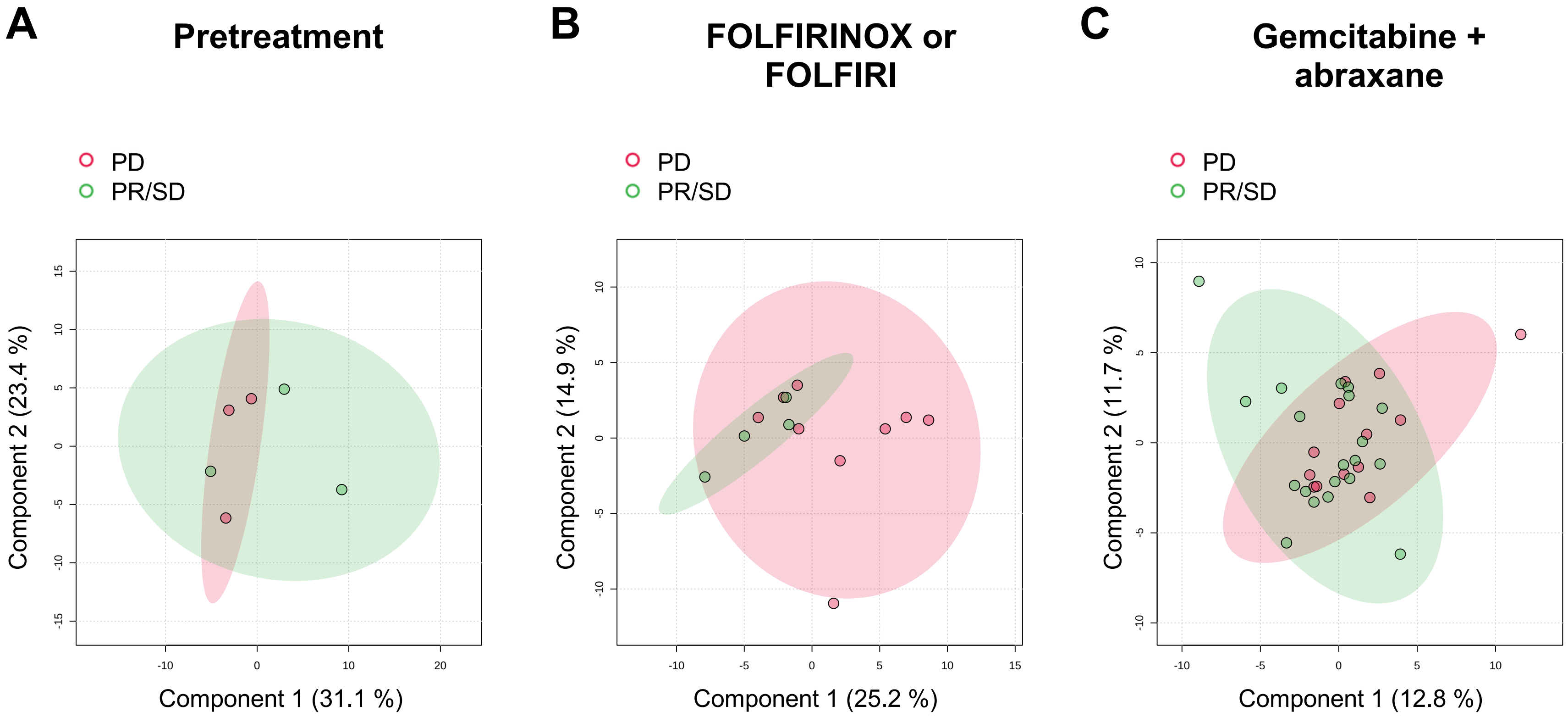

Supplementary Figure S3

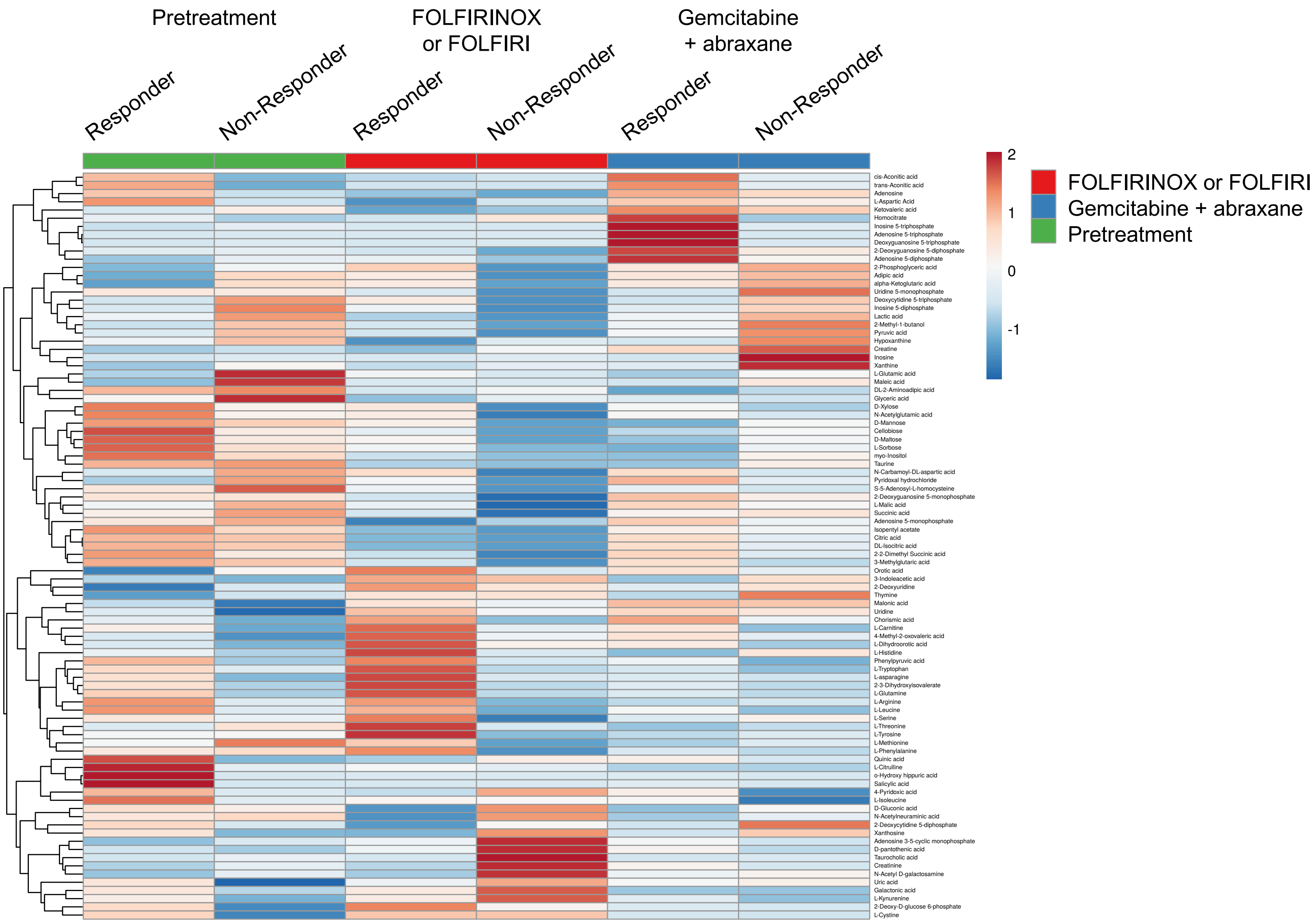

Supplementary Figure S4

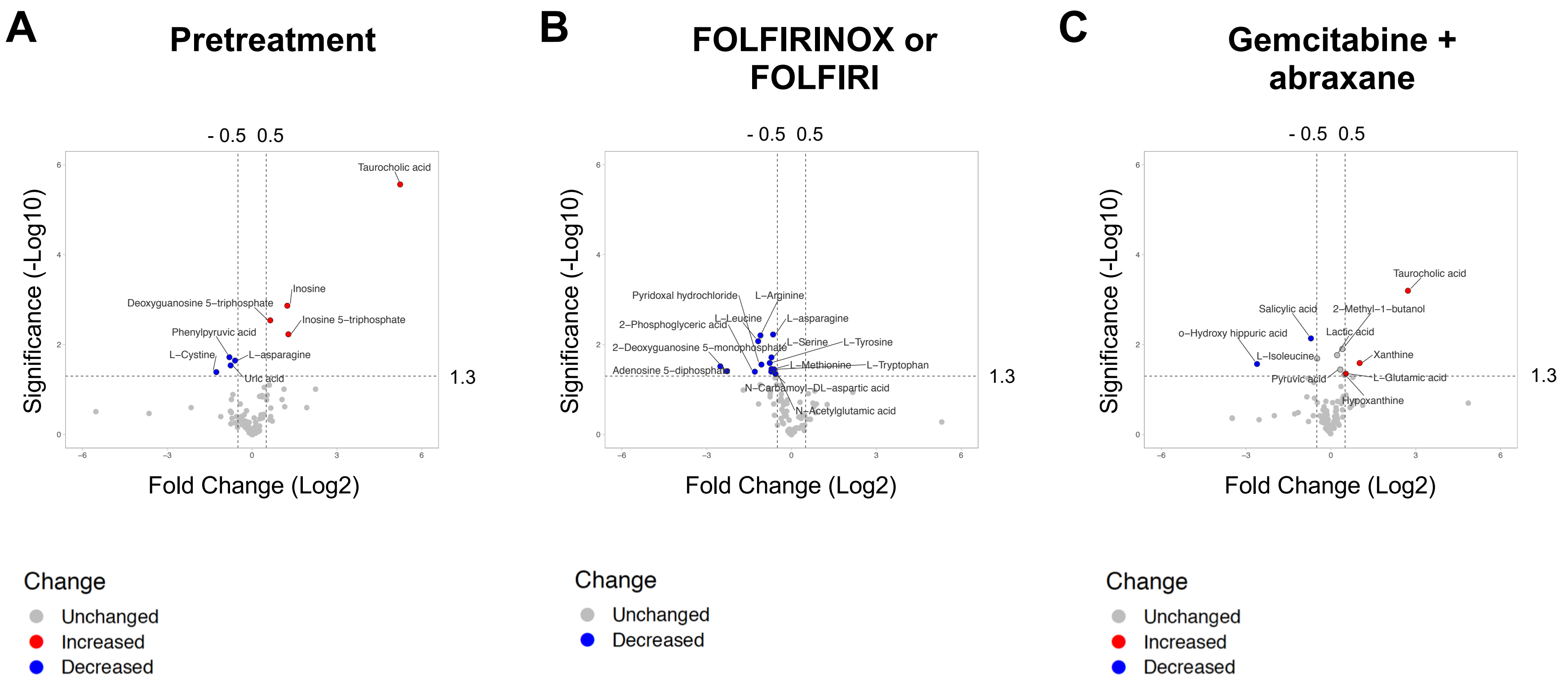

Supplement: Supplementary file 1 [file cancers-15-03020-s001.zip › Supplementary Figures.pdf]
